# Functional and Aesthetic Recovery of Congenital Muscular Torticollis with Intramuscular Stromal Vascular Fraction Enriched Fat Grafting

**DOI:** 10.7759/cureus.975

**Published:** 2017-01-12

**Authors:** Juan Monreal

**Affiliations:** 1 Plastic Surgery, Hospital Moncloa

**Keywords:** fat grafting, fat, congenital muscular torticollis, stromal vascular fraction, sternocleidomastoid muscle, lipofilling

## Abstract

Congenital muscular torticollis is a well-known pathological condition caused by the contracture and shortening of the sternocleidomastoid muscle. This condition is manifested by a neck postural deformity often accompanied by some kind of facial asymmetry. Once diagnosed, treatment by early physiotherapy is generally successful in a high percentage of patients if performed during the first year of life. Later, especially after the fourth year, conservative treatment is usually far less effective, and surgical techniques remain the only way to improve neck contour and function. The author reports two cases of adult patients affected by this condition and successfully treated with a novel therapeutic approach consisting of percutaneous myotomies and intramuscular cell-assisted fat grafting.

Two cases of adult patients diagnosed with congenital muscular torticollis were analyzed after treatment with percutaneous myotomies and intramuscular fat grafting. The first patient had a history of unsuccessful treatment in infancy with bipolar release of the sternocleidomastoid muscle and was treated with two sessions of fat grafting. The second patient had a history of neglected torticollis and was treated with a single session of cell-assisted fat grafting. In both cases, facial asymmetries were simultaneously treated with the same fat grafting protocol used to treat the muscle. Improvements in muscle function and in face and neck contours were extremely good and stable in both patients. The postoperative course for both patients was uneventful and with a very short and easy recovery when compared with the techniques described to date.

Neglected congenital muscular torticollis in adults, or in patients who have not responded adequately to surgical treatment, has been treated safely with percutaneous myotomies and intramuscular fat grafting. The benefit is a scarless technique that provides simultaneous recovery of neck aesthetics and muscle function together with a very short recovery time. Further studies must be conducted to properly evaluate the long-term safety and convenience of cell enrichment to achieve better and long-lasting results.

## Introduction

Congenital muscular torticollis (CMT) is a term used to define a pathological condition that courses with a shortening and contracture of the sternocleidomastoid muscle (SCM) causing a postural deformity of the neck and face. It is the most common cause of torticollis in children and the most common musculoskeletal anomaly after club foot and congenital hip dysplasia, with an incidence of 0.3%–2% of newborns [1-2]. If left untreated, the patient will usually develop various degrees of facial asymmetry with the flattening and recession of the malar, mandibular, and frontal areas accompanied by orbital dystopia. Indeed, several degrees of plagiocephaly were detected in 80­­%–90% of cases and was more severe and frequent in untreated patients.

**Figure 1:**
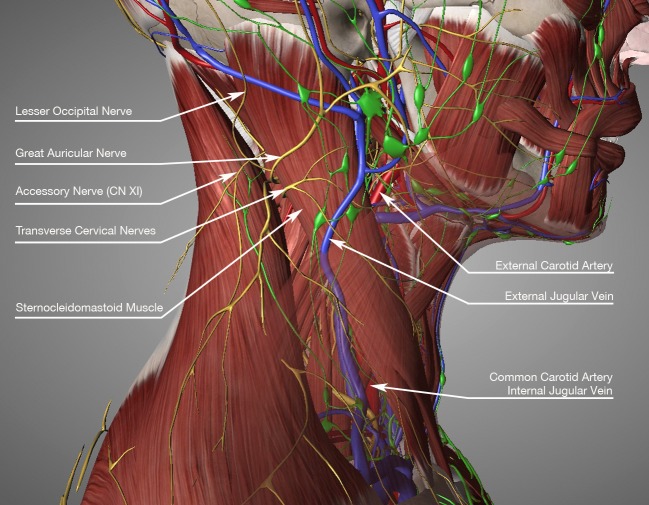
Sternocleidomastoid muscle anatomy

The SCM is a paired striated muscle obliquely crossing the sides of the neck and covered by the two layers of the deep cervical fascia (Figure 1).

From the surgical point of view, the most important relationships are deeper with the carotid artery, internal jugular vein, lymphatic vessels and nodes, and the spinal accessory nerve (XI), which crosses the muscle at its superior third, emerging from its posterior border and supplying innervation to the muscle. Deep branches of the second and third cervical nerves provide additional innervation. The arterial supply is segmental and comes from the occipital artery (upper third), branches from the superior thyroid artery (middle third), and branches from the thyrocervical trunk (lower third). Of all the standard techniques described in the literature, endoscopic muscle transection is the only one that can put at risk the great auricular and spinal nerves. The other techniques can put into partial risk the vascular supply and the greater auricular nerve.

Management of these patients differs according to the time when the diagnosis was established. Conservative treatment with physical therapies is usually successful in 70%–90% of patients, especially if performed during the first year of life [3]. Between the second and fourth years, conservative treatment quickly loses effectiveness, making a surgical treatment plan more appropriate. From the fifth year, surgery in its different forms is the only effective way of treatment, although in adulthood, surgical treatment is associated with more failures and relapses.

The purpose of this article is to report the author’s experience in the treatment of two cases of CMT in adults with a novel therapeutic approach not described previously and based on percutaneous myotomies and simultaneous fat grafting. The rationale for this approach is to simultaneously improve muscle volume, contours, and dynamics by releasing internal fibrosis and padding the released muscle with autologous fat.

## Case presentation

Both patients rejected standard surgical treatments, were informed about the objective and limitations of the proposed procedure, and signed the corresponding informed consent statement. Both patients were treated under general anesthesia in a supine position and following the same surgical protocol, which consisted of percutaneous myotomies to release internal fibrosis and the simultaneous padding of myotomy sites with fat parcels. Facial asymmetries were treated during the same operative procedure and with the same type of fat grafting protocol applied to the muscle. Preoperative antibiotic prophylaxis with cephalexin 2 gr and dexamethasone 8 mgr intravenous (IV) was used in both patients.

The first step was the harvesting of the estimated amount of fat tissue needed to perform the padding of myotomy sites and the correction of facial asymmetries. For both patients, tissue was harvested from abdominal fat deposits using 3 mm multi-hole cannulas, as described in a previous study by the author [4], and was processed by washing with lactated Ringer’s solution and decanting for at least 20 minutes. For the second patient, an additional 175 ml of fat tissue was collected and processed to obtain the stromal vascular fraction (SVF) component. The adipose tissue was rinsed with lactated Ringer’s solution and dissociated at 38°C for 40 minutes using Type I collagenase at a concentration of 250 collagen digestion units (CDU) per ml of final volume. The final volume is the volume of the adipose tissue plus an equal volume of lactated Ringer’s solution. The digested tissue was centrifuged at 800 × g for 10 minutes. The supernatant fluid was discarded after centrifugation, and the pellet was then resuspended and washed three times with lactated Ringer's solution. Cell counting was performed with an Adam-MC (NanoEnTek, Seoul, Korea) giving a yield of 670,000 viable cells/gr of lipoaspirate. 125 ml of fat tissue was mixed with SVF cells to a final ratio of 930,000 SVF cells/ml of fat graft.

**Figure 2:**
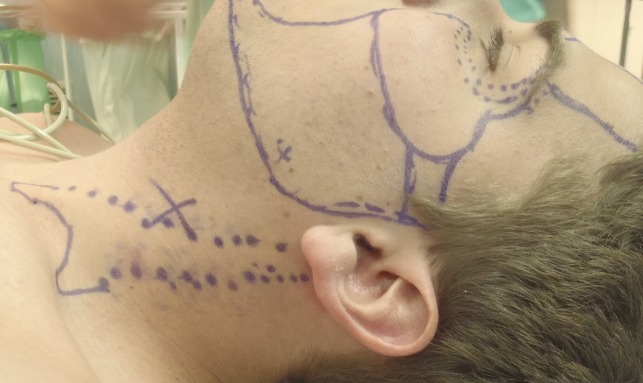
Topographic landmarks of SCM before surgical treatment

With the patients in a supine position and head turned to the opposite side to tighten the affected SCM, an accurate map of the fibrotic areas of the muscle was made (Figure 2).

**Figure 3:**
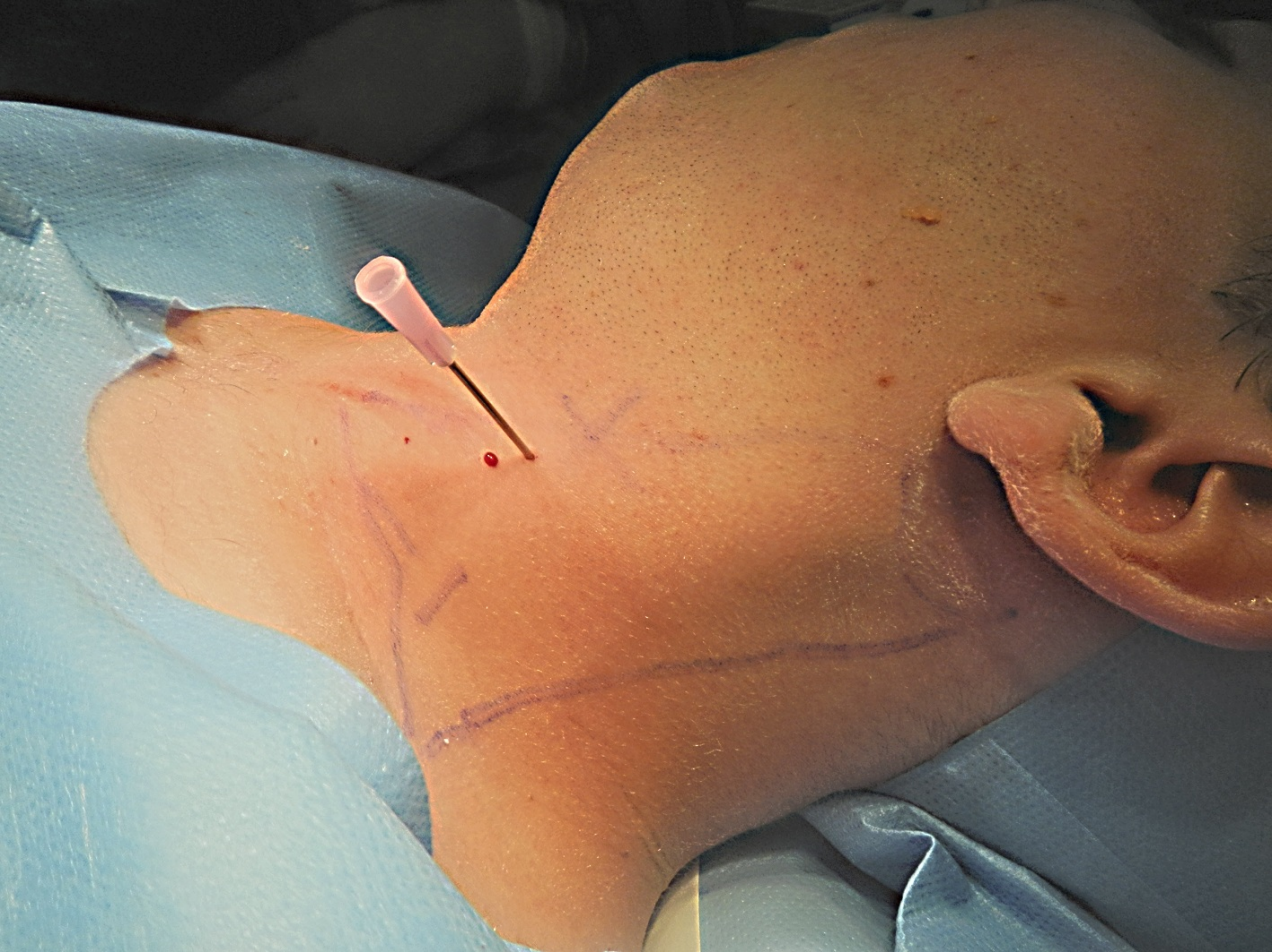
Intraoperative view showing an 18 G needle used to perform myotomies Myotomies were performed percutaneously with an 18 G needle all along the muscle length and thickness.

**Figure 4:**
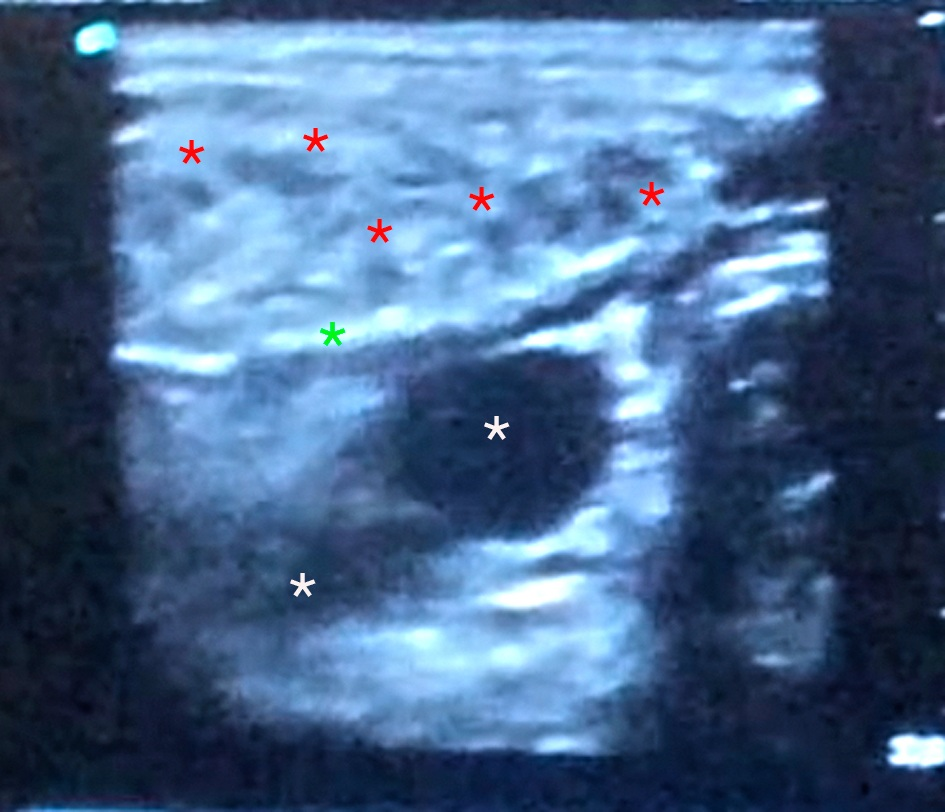
Intraoperative ultrasound image Red asterisks mark fat grafting depots. White asterisks mark the internal carotid artery and vein. The green asterisk marks the posterior fascia of the SCM.

**Figure 5:**
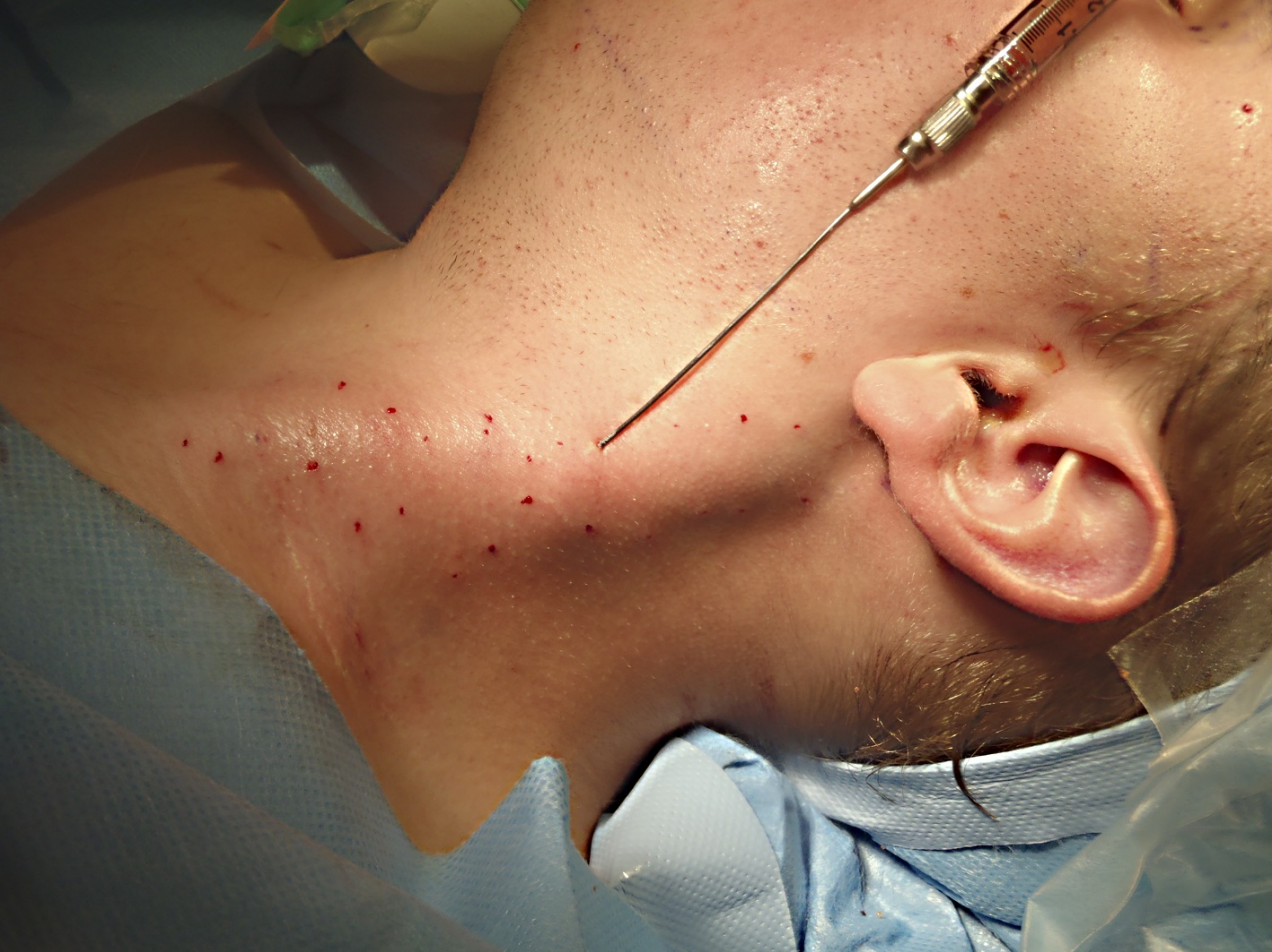
Intramuscular injection of fat parcels Fat grafting was performed at the same time as the needle myotomies or as an additional step using a 1.4 mm blunt cannula.

The topographic landmarks of spinal accessory and greater auricle nerves and carotid artery were marked as well. With a 18-gauge needle, multiple percutaneous myotomies were performed all along the muscle length and thickness, being more aggressive in areas with more intense fibrotic changes (Figure 3). To avoid damage to deep vessels and in an attempt to treat the whole muscle thickness, hot areas were treated under ultrasound guidance (Figure 4). Depending on the degree of fibrosis, padding of myotomy sites with fat parcels was performed at the same time as needle insertion or as an additional step with a 1.4 mm blunted tip cannula (Figure 5). The average amount of fat injected was 0.01 ml per myotomy site. The final point to stop the myotomy-graft process was reached when the entire muscle length and width was treated and the degree of softening in the fibrotic areas was considered as acceptable.

Patients were discharged the day after surgery with no limitation on daily activities except for those requiring exercises or ample neck movements. No collars were used. After the first week, patients were allowed to move their necks freely. During the first two postoperative months, both patients were encouraged to perform physical exercises at home to improve muscle elongation and range of motion and to reeducate posture.

### Case 1

**Figure 6:**
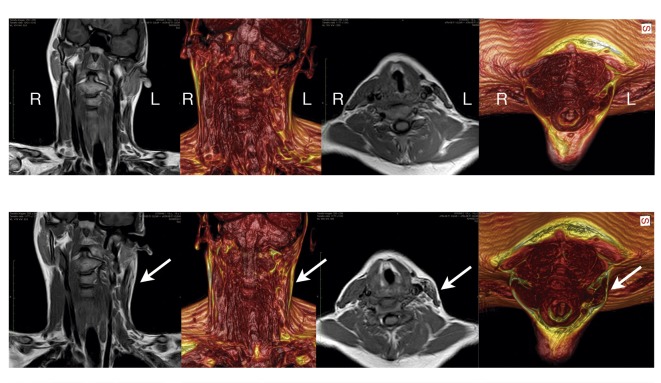
Preoperative and postoperative MRI of Case 1 Upper row: preoperative MRI images. Lower row: postoperative MRI images at six months (arrows point out treated muscle).

A 19-year-old male patient who had been treated in infancy for CMT presented with a 16-year history of neck deformity affecting the left SCM. The primary procedure involved the bipolar release of both muscular insertions and simultaneous z-plasty of sternal insertions; the patient reported limited and transient improvement that worsened over the following years. During the physical exam, a static lateral tilt to his left side and a deviation of the chin to the right side were seen. The patient showed signs of facial dysplasia with a flattening of the ipsilateral malar mound and mandibular angle, orbital dystopia, and a recession of the left frontal area. The scars of the previous surgery were located on the mastoid and the sternoclavicular regions, under which scar tissue was palpable and was more evident in the distal insertions. Magnetic resonance imaging (MRI) (Figure 6) revealed muscular sclerosis and volume deficits in the affected muscle.

**Figure 7:**
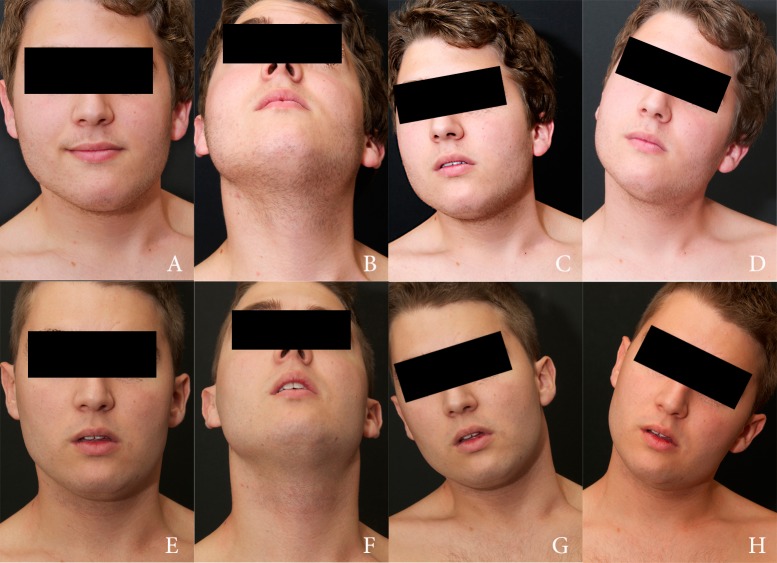
Preoperative and postoperative views of Case 1 Preoperative images of Case 1 (A to D) and postoperative images (E to H) 18 months after two sessions of micromyotomies and fat grafting.

During the first session, 45 cc of adipose tissue was used to treat asymmetric facial areas (left frontal, malar, and mandibular); needle micromyotomies and an additional 35 cc were used to treat the SCM. During the second session, needle micromyotomies and a total of 30 cc of adipose tissue were used to treat SCM only. At the end of each session, no bandages nor collars or any other means of cervical stabilization were used. The patient was encouraged and instructed to perform postural and muscle stretching exercises beginning one week after each procedure. No other physical or orthotic therapy was used. A new MRI exam was performed during the sixth postoperative month in order to check the status of the affected muscle and visualize its inner structure (Figure 6). Figure 7 shows preoperative images and postoperative results at 18 months.

### Case 2

**Figure 8:**
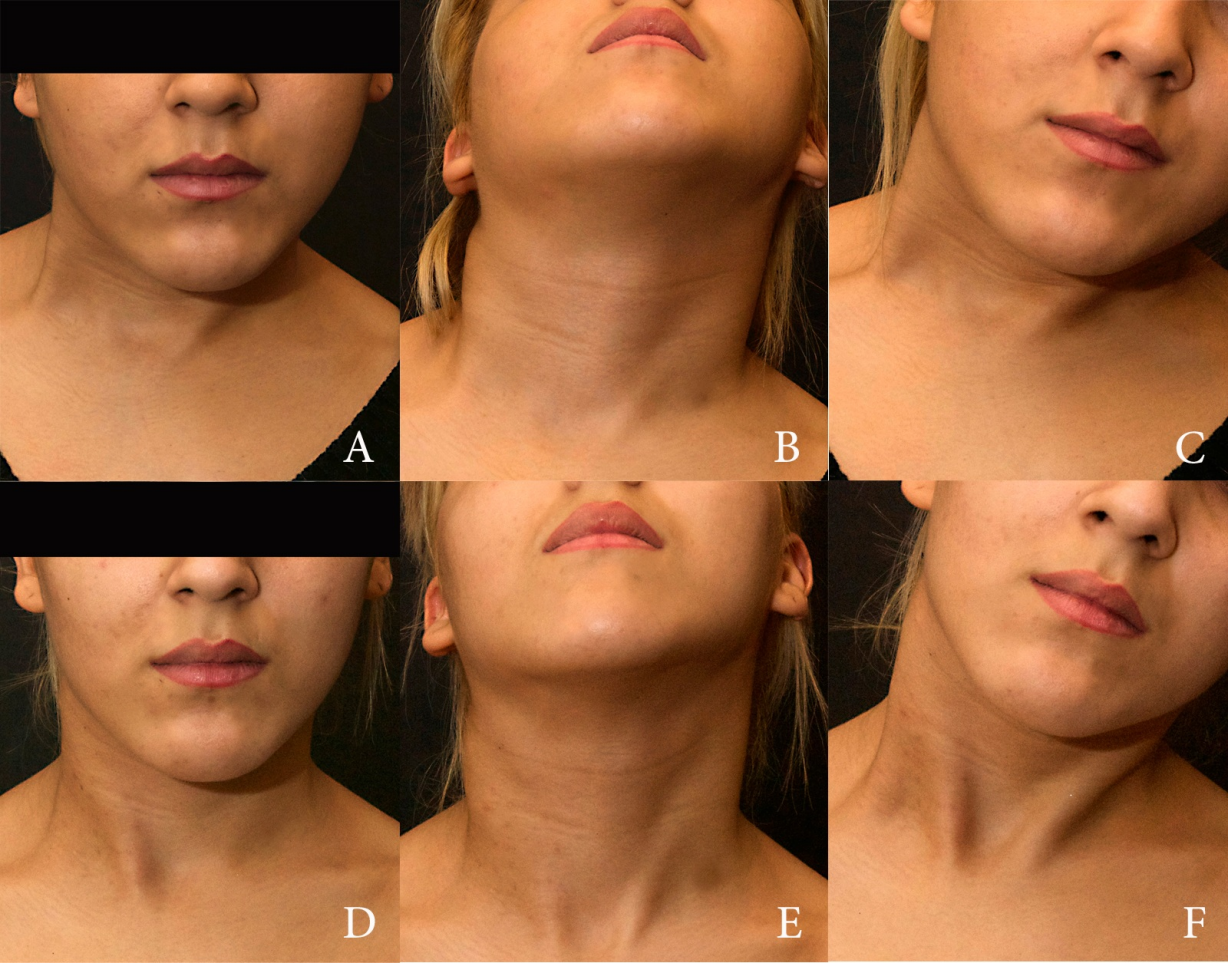
Preoperative and postoperative views of Case 2 Preoperative views of Case 2 (A to C) and postoperative views six months (D to F) after a single session of SVF enriched fat grafting.

A 23-year-old female presented with a history of neglected CMT. The patient presented with a severe contracture and atrophy of her right SCM with concomitant muscular imbalance of scapular waist and craniofacial asymmetry affecting the right frontal, malar, and mandibular regions as well as orbital dystopia. There were no antecedents of physical therapy or surgeries. Due to financial issues, it was not possible to obtain preoperative or postoperative MRI exams. In an attempt to obtain the best performance from a single procedure, the surgical plan included a thorough micromyotomies mapping of the whole muscle mass (including proximal and distal insertions where most residual fibrosis was detected). Padding of myotomy sites and facial asymmetry correction were performed with cell-assisted fat grafting protocol as described above. A total of 125 cc of enriched fat were used – 45 cc to treat SCM and 80 cc to treat facial asymmetries. Figure 8 shows preoperative images and the postoperative result after six months.

### Results

The early postoperative course after surgery in both patients was uneventful. SCM inflammation was classified as normal or limited and did not require drug therapy after the first 48 hours. Basic postural exercises and active stretching exercises were encouraged from the first postoperative week, and both patients resumed their normal daily activities between the seventh and tenth postoperative day. During the first month after the first surgery, the first patient reported significant symptomatic improvement in tightness; the improvement gradually increased over the following months. Before the second surgery (six months postoperatively), the patient's improvement in terms of mobility and postural attitude was rated by the patient as satisfactory but incomplete. Six months after the second surgery (12 months after the first), the postural attitude of the patient, as well as his facial and neck symmetry, were rated as excellent (0° deviation from the midline), with neck rotation and lateral flexion almost complete and unrestricted. The stability of the results could be observed until the 18th postoperative month. Regarding the second patient, improvements in range of motion and postural attitude were achieved earlier; and at two months postoperatively, only some disbalance in scapular waist remained pending on reeducation.

## Discussion

Reports of CMT treatment in the plastic and aesthetic surgery literature are scarce, although the vast majority of adult patients seek treatment for aesthetic reasons. Despite an extensive bibliography search, the author was not able to find any modern reference to a percutaneous approach to torticollis treatment. Interestingly, during the Middle Ages, some traveling practitioners could have been performing percutaneous tenolysis to treat this condition. Cheselden in 1749 was the first to describe an open tenotomy procedure to release clavicular insertions of the SCM. With some technical variants, this surgical approach continues to be the most popular procedure for the treatment of CMT.

Several open and endoscopic surgical techniques to release SCM have been described in the medical literature, all of which aimed to improve static and dynamic neck appearance and function. Large series have demonstrated that surgery is the most satisfactory treatment for patients older than one year of age, with the best outcomes in patients between one and four years.

The surgical techniques described in the medical literature [5-9] range from bipolar release of mastoid and sternoclavicular insertions, release and lengthening by z-plasties of one or both muscle insertions, or subperiosteal endoscopic elongation and muscle transection. Open releases, with or without the associated z-plasty, are performed through skin incisions placed near the sternoclavicular and mastoid muscle insertions. Of all the above techniques, endoscopic transection is maybe the one that requires a higher learning curve and has a higher risk of complications due to damage to the greater auricular nerve and/or the spinal nerve. In severe cases of CMT with severe fibrosis and shortening, complete removal of SCM has also been described using fat grafting in a second operative procedure to simulate muscle contours [10]. The aim and purpose of all the aforementioned techniques is to achieve muscular elongation to improve the static and dynamic balance of the neck (without causing loss of strength) and to prevent worsening of facial asymmetry and the appearance of secondary disorders such as cervical scoliosis. The ultimate success of each of these techniques depends mainly on the age of treatment, the severity of the case, and the technique used. In different retrospective studies, it has been estimated that a successful outcome occurs in 81%–89% of patients, and there is an estimated 11% incidence of recurrence following these techniques, especially when it comes to adult patients. Usually, physical therapy and/or orthotic measures must be resumed in the postoperative period to prevent recurrence of scar contracture. Successful outcomes with the aforementioned techniques will provide improvements in muscle length and range of motion but will not improve its internal structure and the degree of fibrosis. Additionally, the side-to-side symmetry of neck contours and volume is not always improved. There is only one description in medical literature on the use of fat grafting as an adjunct to improve neck contour after complete removal of the entire muscle.

Intramuscular fat grafting is a relatively common approach to treat different contour defects of the body. It has been used extensively to augment buttocks, calves, or breast areas with excellent results and few side-effects if properly performed. The rationale in the use of fat grafting in the treatment of CMT is to improve, with a scarless technique, all the defects that affect the SCM, particularly in adult cases – that is, to try to obtain true tissue regeneration. With multiple percutaneous needle myotomies, contracture and internal fibrosis are freed all along the muscle and not only in areas of muscle insertion. Adipose tissue and its SVF component were used simultaneously as a spacer and as a possible source of tissue regeneration, although this should be demonstrated in future cases with more data. The simultaneous recovery of muscle volume offers a better cosmetic result improving the symmetry and topography of the neck. These benefits are coupled with a rather quick and easy postoperative period and the absence of scars. The author still recommended a course of physical exercises at home to both patients as a means to recover the plasticity of the affected muscle and to reeducate a long-lasting deficient posture.

## Conclusions

After an exhaustive literature review, it can be concluded that this is the first description of intramuscular fat grafting in the treatment of CMT to date. The open and endoscopic techniques described to date have demonstrated good results in adults in terms of the range of motion of the neck and secondary craniofacial deformities. The benefits of the procedure described, with respect to open techniques, are the scarless integral rehabilitation of the SCM through an improvement in its dimensions and inner structure as well as an easier and shorter postoperative course. Although a simple fat grafting technique was useful to obtain a good result, SVF enriched fat grafting provided comparable results earlier and in a single step. Further studies are needed to assess the stability of the results in the long term and the possibility of real muscle regeneration produced by the SVF component of adipose tissue.
